# MixMC: A Multivariate Statistical Framework to Gain Insight into Microbial Communities

**DOI:** 10.1371/journal.pone.0160169

**Published:** 2016-08-11

**Authors:** Kim-Anh Lê Cao, Mary-Ellen Costello, Vanessa Anne Lakis, François Bartolo, Xin-Yi Chua, Rémi Brazeilles, Pascale Rondeau

**Affiliations:** 1 The University of Queensland Diamantina Institute, The University of Queensland, Translational Research Institute, Brisbane, QLD, Australia; 2 Institut de Mathématiques de Toulouse, UMR CNRS 5219 INSA Université de Toulouse, Toulouse, France; 3 Queensland Facility for Advanced Bioinformatics, The Institute for Molecular Bioscience, Brisbane, QLD, Australia; 4 Danone Nutricia Research, Palaiseau Cedex, France; Wilfrid Laurier University, CANADA

## Abstract

Culture independent techniques, such as shotgun metagenomics and 16S rRNA amplicon sequencing have dramatically changed the way we can examine microbial communities. Recently, changes in microbial community structure and dynamics have been associated with a growing list of human diseases. The identification and comparison of bacteria driving those changes requires the development of sound statistical tools, especially if microbial biomarkers are to be used in a clinical setting. We present mixMC, a novel multivariate data analysis framework for metagenomic biomarker discovery. mixMC accounts for the compositional nature of 16S data and enables detection of subtle differences when high inter-subject variability is present due to microbial sampling performed repeatedly on the same subjects, but in multiple habitats. Through data dimension reduction the multivariate methods provide insightful graphical visualisations to characterise each type of environment in a detailed manner. We applied mixMC to 16S microbiome studies focusing on multiple body sites in healthy individuals, compared our results with existing statistical tools and illustrated added value of using multivariate methodologies to fully characterise and compare microbial communities.

## Introduction

The human gut microbiome contains a dynamic and vast array of microbes that are essential to health and provide important metabolic capabilities. Until recently, studying these complex communities has been difficult and generally limited to classical phenotypic techniques [1, 2]. With the improvement of high-throughput sequencing technology, the ability to profile complex microbial communities without the need to individually culture organisms has increased dramatically. These sequencing methods range from RNA sequencing (RNA-seq), chromatin immunoprecipitation sequencing (ChIP-seq), metagenomic and 16S rRNA gene amplification analysis of microbial populations. 16S rRNA sequencing in particular has substantially changed our understanding of phylogeny and microbial diversity, and is quickly becoming a staple for profiling microbial communities and their abundances from soil to humans. With this sequencing technique, hypervariable regions within the gene are amplified, sequenced, and clustered into operational taxonomic units (OTU). Taxonomic classification of representative sequences from each cluster is then aligned against a database of previously characterised 16S ribosomal DNA reference sequences to identify bacteria of interest. As alterations and changes in microbiomes have been associated with a range of diseases including obesity [3–5], Crohn’s disease [6] or ankylosing spondylitis [7], it is integral that we analyse this data appropriately given the impact on human health and disease treatment outcomes [8].

A number of statistical analysis tools have been proposed to examine differences between microbial communities as well as to identify features that are key to driving the differences. Those methods were developed to accommodate the specific *sparse* nature of microbiome data. White *et al*. proposed Metastat, a non parametric t-test based on permutation or a Fisher’s exact test when data are sparsely sampled [8]. Their approach was a first step towards identifying organisms whose differential abundance correlated with disease. Paulson *et al*. developed a zero-inflated Gaussian (ZIG) distribution mixture model to account for biases due to undersampling of the microbial community [9].

The other characteristic of microbiome data is their underlying *compositional* structure. Due to varying sampling/sequencing depths between samples from high-throughput sequencing, each OTU count is converted into relative abundance (proportion) in each sample. This intuitive pre-processing step results in compositional data which reside in a simplex sample space rather than the Euclidian space [10]. As a consequence, conventional statistical methods including correlation coefficients or univariate methods may lead to spurious results as the independence assumption between predictor variables is not met [11–13]. A growing list of references advocate against the use of such methods for microbiome compositional data [14, 15]. One solution that was proposed by Aitchison is to transform compositional data into Euclidian space using centered log ratio transformation (CLR) before applying standard univariate or multivariate methods [10, 14, 16].

Another important aspect to consider when analysing microbiome data is that microbial communities modulate and influence biological pathways as a whole. Therefore univariate statistical approaches that test each OTU feature individually, disregarding interactions or correlations between features may provide limited insight into the microbiome. One could instead consider multivariate methods as they analyse the entire set of OTUs at once. So far, most multivariate approaches are solely used to visualise diversity patterns, such as unsupervised Principal Coordinate Analysis (PCoA [17]) based on sample-wise distance/dissimilarity matrices to scale for species abundance (e.g. Bray-Curtis [18], unweighted [19] or weighted Unifrac [20] distances), or supervised between-class analysis [21] to segregate sample groups. However, those multivariate approaches limit our understanding as they do not indicate which key species discriminate the sample groups, with the exception of ALDex2 [15], and LEfSe [22]. Those methods still rely on univariate tests (Welch’s t- or Wilcoxon rank test) as a first step to assess the significance of each OTU.

Finally, the other critical issue we address in this study is high inter-subject variability [4], which is often reduced with an appropriate experimental repeated-measures design where each subject acts as its own control. Thus, microbial sampling is performed repeatedly on the same subjects over different habitats. While such experimental design has been widely adopted by community profiling studies such as the Human Microbiome Project (HMP, [23, 24]) to define a ‘healthy’ microbiome community by characterising different body sites in the same subjects, very few statistical methods have taken advantage of this design and accommodate inter-subject variability.

We introduce mixMC, a multivariate analysis framework for 16S data to identify OTU features discriminating multiple groups of samples. mixMC addresses the limitations of existing multivariate methods for microbiome studies and proposes unique analytical capabilities: it handles compositional and sparse data, repeated-measures experiments and multiclass problems; it highlights important discriminative features, and it provides interpretable graphical outputs to better understand the microbial communities contribution to each habitat. We applied mixMC to multiple body site studies in healthy individuals from HMP and the study from Koren *et al*. [25], compared our results with existing univariate statistical approaches and provided thorough interpretations of the microbial communities unraveled using our multivariate analyses.

## Material and Methods

**Fig 1:**
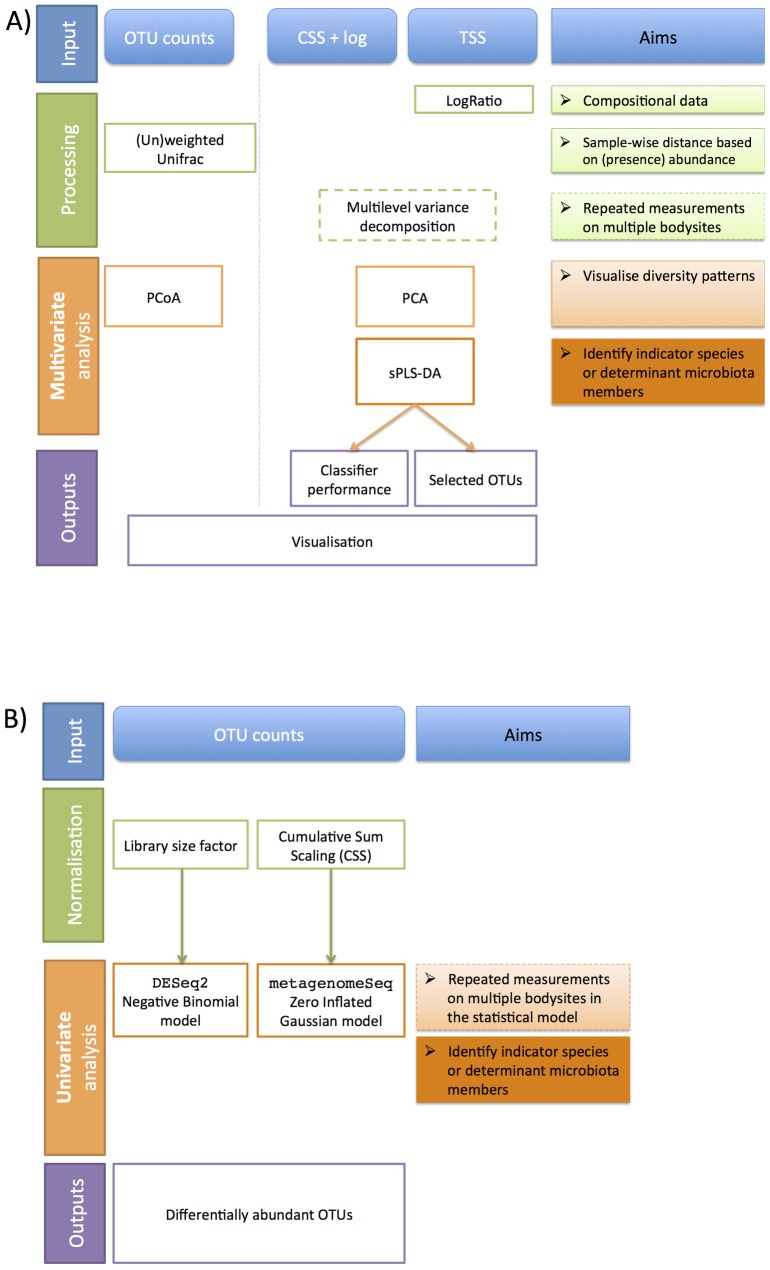
Comparison between multivariate and univariate statistical analysis frameworks for 16S microbiome data. **(A)** Multivariate mixMC framework including processing/normalisation, optional repeated measures design, unsupervised and supervised analyses, **(B)** Univariate framework, including normalisation and optional repeated measures design analysis.

We analysed publicly available 16S data from the NIH Human Microbiome Project and cross-compared our results with the microbiome study from Koren *et al*. [25]. The data were processed by the open-source bioinformatics software QIIME [26] for the 16S variable region 1–3. We first describe the different processing, and normalisation steps, and the statistical methods applied in this study, summarised in Fig 1A.

### Data processing and normalisation

One of the characteristics of 16S data is their sparse nature and the differences in sequencing depth, which makes preprocessing and normalisation steps crucial when the aim is to characterise and differentiate microbial communities. Since this study focuses only on beta diversity and differences in abundance between sample groups, we do not recommend using a rarefaction step prior to the mixMC analyses.

#### Prefiltering

Bokulich *et al*. demonstrated that strict quality filtering of reads greatly improves measures for microbial community profiling [27]. After removing samples with a very low number of total OTU counts (less than 10), we removed OTUs with proportional counts across all samples below 0.01%. While this may appear drastic, this prefiltering step can counteract sequencing errors, estimated to be 1/1000 in Illumina MiSeq for example [2, 28]. The prefiltering step avoids spurious results in the downstream statistical analysis. The proposed threshold is the default value in QIIME that was also used in other microbiome studies (e.g. [29, 30]).

#### Normalisation

Normalisation must address the issues of sparse counts and differences in sequencing depth and needs to be carefully chosen as this step can strongly affect the downstream statistical results [9]. So far, two types of normalisations have been proposed for microbiome studies.

The commonly used Total Sum Scaling normalisation (TSS) divides each OTU count by the total number of counts in each individual sample to account for uneven sequencing depths across samples. However, since TSS reflects relative information (i.e. proportions), the resulting normalised data reside in a simplex rather than an Euclidian space which may lead to spurious false discoveries if standard statistical methods are applied [10]. The solution is to transform TSS data to project them to the Euclidian space using log ratio transformations. The Centered Log Ratio transformation (CLR) has been recently applied in several compositional data studies [14–16]. Let ***x*** = (*x*_1_, ⋯, *x*_p_)′ denote a composition on the *p* TSS normalised OTU counts, then the CLR transformation is defined as

$$\begin{array}{c} y={\left(y_{1},\cdots ,y_{p}\right)}^{\prime }={\left(log\frac{x_{1}}{\sqrt[{p}]{\prod_{i=1}^{p}x_{i}}},\cdots ,log\frac{x_{p}}{\sqrt[{p}]{\prod_{i=1}^{p}x_{i}}}\right)}^{\prime }. \end{array}$$

Alternatively, the Cumulative Sum Scaling normalisation (CSS, [9]) was developed to prevent TSS bias in differential abundance analysis with sparse counts. CSS can be considered as an extension of the quantile normalisation approach and consists of TSS scaling raw counts that are relatively invariant across samples, up to a percentile determined using a data-driven approach. CSS therefore partially accounts for compositional data. We applied CSS on the log transformed counts using the metagenomeSeq package [31].

### Methods

The main objective of our study is to extend and apply multivariate statistical analysis methods for microbiome compositional data. The mixMC framework (Fig 1) includes unsupervised analyses to visualise diversity patterns with Principal Component Analysis (PCA) and supervised analyses to identify indicator species or determinant microbiota members characterising differences between habitats or body sites (sparse Partial Least Square Discriminant Analysis, sPLS-DA). In addition, our framework addresses a commonly encountered experimental design in microbiome studies called *repeated-measures design*, where microbial sampling is performed on the same individuals but in different body sites to detect differences between habitats. This design leads to analytical challenges in order to be able to discern subtle differences *between* body sites from the large variation between individuals *within* each body site.

#### Unsupervised multivariate analysis

PCA variants, such as Principal Coordinate Analysis (PCoA, [17]) allows for dimension reduction of the data and visualisation of diversity patterns in microbiome studies. PCoA is commonly applied to non Euclidian sample-wise dissimilarity matrices (e.g. Bray-Curtis [18]) or phylogenetic distances between sets of taxa in a phylogenetic tree (weighted or unweighted Unifrac distance, [19, 20]). Alternatively, and to avoid spurious results arising from compositional data PCA can be applied on log ratio compositional data using either CLR transformation, or Isometric Log Ratio transformation (ILR, [32], described in S1 Text). In mixMC we applied PCA on ILR transformed data using customised R scripts from the robCompositions package [33].

#### Multilevel variance decomposition

One way to account for repeated measurements designs is to separate body site variation (termed ‘*within variation*’) from individual variation (termed ‘*between subject variation*’) via variance decomposition. In univariate analyses, this step refers to repeated measures ANOVA (also called within-subjects ANOVA). In multivariate analysis we refer to ‘multilevel approach’ [34]. The within subject variation is obtained by calculating the net differences between repeated observations (i.e. between each body site within each individual). Since the within subject variation assesses the difference in the body sites within each subject and disregards the possibly large individual variation, the within variation can then be used as input data in the subsequent multivariate statistical analysis [35]. In mixMC, the multilevel variance decomposition is applied on the log ratio transformed data described above, prior to the multivariate analyses (Fig 1A). Note that the variance decomposition in the multilevel approach does not take into account the correlation structure or order between measurements and is not appropriate for a time course experiment where the objective is to examine the effect of time in a study (see for example applications of linear mixed model splines for those specific cases [31, 36]).

#### Supervised multivariate analysis

The multivariate approach sparse Partial Least Squares Discriminant Analysis (sPLS-DA, [37]) is an extension of the PLS algorithm from Wold *et al*. [38] to perform feature selection with multilevel decomposition [35]. In mixMC we further extended the multilevel sPLS-DA for microbiome data using either CSS normalised data, or TSS+CLR data.

*Principle of PLS-DA*. PLS-Discriminant Analysis is a multivariate regression model which maximises the covariance between linear combinations of the OTU counts and the outcome (a dummy matrix indicating the body site of each sample). Covariance maximisation is achieved in a sequential manner via the use of latent component scores. Each component is a linear combination of OTU counts and characterises a particular source of co-variation between the OTU and the body sites. As a consequence, the final number of components summarising most of the information from the data must be specified. The sparse version of PLS-DA, sPLS-DA uses Lasso penalisations [39] to select the most discriminative features in the PLS-DA model. The penalisation is applied componentwise and the resulting selected features reflect the particular source of covariance in the data highlighted by each PLS component.

*Parameters and performance evaluation*. The number of features to select per component must be specified in sPLS-DA and is usually optimised using cross-validation. In this study we used 10-fold cross-validation repeated 100 times. For varying features selected by sPLS-DA the classification error rate resulting from the cross-validation process was then recorded and the lowest error rate indicated the optimal number of features to select on each component. This procedure concurrently indicated the optimal number of components for the sPLS-DA model. Once those parameters chosen, the final sPLS-DA model was run on the entire data set to obtain the final list of discriminative OTUs for each component.

*Graphical and numerical outputs*. We further characterised each selected OTU by calculating its median normalised count in each body site. An OTU was defined as ‘contributing to a body site’ if the median count in that specific body site was higher than in any other body site. We graphically represented the contribution of each selected OTU with a barplot where each OTU bar length corresponds to the importance of the feature in the multivariate model (i.e. the multivariate regression coefficient with either a positive or negative sign for that particular feature on each component) ranked by decreasing importance starting from the bottom, and with colours matching the contributing body site. The contribution plot can display the bacterial taxonomy at any specified level, here we chose the family level. We also used circular representations of taxonomic trees using the GraPhlAn software tool [40] to complement the contribution plot with taxonomy information. In this plot the background colour indicates the body sites where the OTU is most abundant, the node size represents the median OTU count in that body site and the node colour indicates a negative (black) or positive (yellow) weight from the sPLS-DA regression coefficient. Other insightful outputs include sample representation where each individual is projected onto the sPLS-DA components, the list of OTU features selected on each component, the cross-validation error rate per component and the number of features contributing to each body site for each component.

The multilevel sPLS-DA framework is implemented in the R package mixOmics [41] using multilevel decomposition [35]. The cladogram was generated using the GraPhlAn Python code [40]. R codes and tutorials are available on our website www.mixOmics.org/mixMC.

#### Univariate analysis

Unlike multivariate methods, univariate methods test each OTU for differential abundance between body sites. P-values obtained were adjusted for multiple testing using the False Discovery Rate (FDR, [42]) at the 5% significance level. We considered two univariate approaches able to analyse repeated-measures experiments (Fig 1B).

DESeq2 was developed for DNA sequencing read count data where mean and variance for the binomial distribution is estimated for each feature [43]. OTU counts are normalised internally to the method with respect to a library size factor estimation, however, this normalisation does not address the issue of compositional data. For microbiome data analysis DESeq2 has served as a basis of comparison to novel methodological developments [9, 15, 44]. We used mean dispersion estimates models as implemented in the R package DESeq2 [45].

ZIG [9] is a mixture model with a Zero-Inflated Gaussian distribution to account for varying depths of coverage that is typical for microbial community under-sampling. In the ZIG model, OTU counts are first log transformed and then CSS normalised (R package metagenomeSeq [31]).

### Case studies

#### HMP case studies

We analysed subsets of the NIH HMP16S data downloaded from http://hmpdacc.org/HMQCP/all/ for the V1–3 variable region. The original data contained 43 146 OTU counts for 2 911 samples measured from 18 different body sites. We focused on the first visit of each healthy individual and further divided the data into two data subsets. For both data sets a preliminary exploratory PCoA confirmed that there was no confounding covariate effect due to run center or gender (see S1 Fig).

*Most diverse body sites dataset*. Understanding microbial community diversity across body habitats is fundamental to study the human microbiome. In their extensive HMP data statistical analysis, Li *et al*. quantified intra-sample diversity using the Shannon index. Based on their results we chose the three most diverse habitats according to all genera-based and OTU-based taxonomic units [46], namely Subgingival plaque (Oral), Antecubital fossa (Skin) and Stool sampled from 54 unique healthy individuals for a total of 162 samples. The prefiltered dataset included 1 674 OTU counts (S1 Table).

*Oral body sites dataset*. While many published analyses have focused on the main microbial habitats (gut, oral cavity, skin and vagina from the [24, 47]), little has been done to comprehensively characterise multiple sites within a single habitat. In this data set we solely considered samples from oral cavity, which has been found to be as diverse as the stool microbiome [46]. The nine oral sites were Attached Keratinising Gingiva, Buccal Mucosa, Hard Palate, Palatine Tonsils, Saliva, Subgingival Plaque, Supragingival Plaque, Throat and Tongue Dorsum. After prefiltering, the data included 1 562 OTU for 73 unique healthy individuals and a total of 657 sample (S1 Table).

#### Koren dataset

Koren and colleagues examined the link between oral, gut and plaque microbial communities in patients with atherosclerosis and controls [25]. We compared our HMP most diverse results to the healthy individuals from this dataset. This study contained partially repeated measures from multiple sites including 15 unique patients samples from saliva and stool, and 13 unique patients only sampled from arterial plaque samples. The data were downloaded from the QIITA database (http://qiita.microbio.me/study/description/349) and included 5 138 OTU. After prefiltering, the data included 973 OTU for 43 samples.

## Results

### Unsupervised analyses on Most Diverse body sites dataset

We applied unsupervised analyses PCoA or PCA on ILR transformed data to visualise diversity patterns between microbial communities, then compared different types of normalisations (TSS-ILR, CSS) followed by a multilevel variance decomposition for repeated measures.

**Fig 2:**
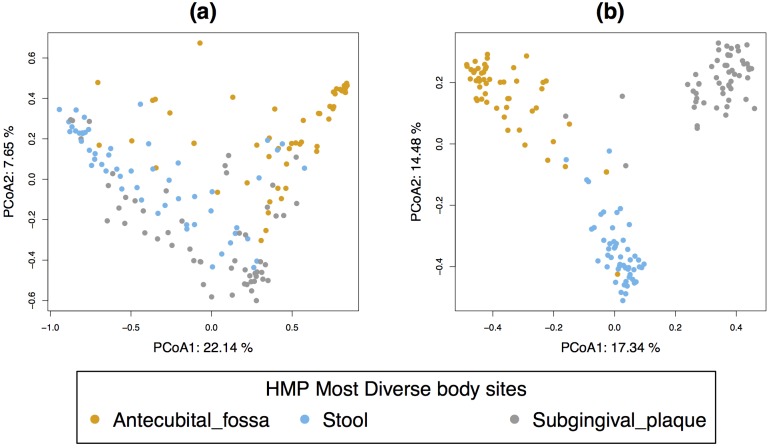
Most diverse data, PCoA sample plots. Sample plot on the first two coordinates with **(a)** weighted Unifrac **(b)** unweighted Unifrac calculated on the filtered OTU count table (based on 1 674 OTU).

A PCoA performed on the filtered OTU raw counts (with no normalisation) showed that the unweighted Unifrac distance could highlight diversity patterns between each body site better than weighted Unifrac (Fig 2). As this study focuses on the most diverse body sites, the presence or absence of microbial communities is expected to drive the differences between body sites more than the relative abundance usually highlighted by weighted Unifrac. Applying PCoA on the unfiltered count data led to similar interpretation (S2 Fig), but we observed a lower amount of explained variance of the first and second coordinate as more ‘noisy’ OTU were present in the data (unweighted Unifrac: 11.28% and 8.95% for the unfiltered data vs. 17.37% and 14.48% for the filtered data).

**Fig 3:**
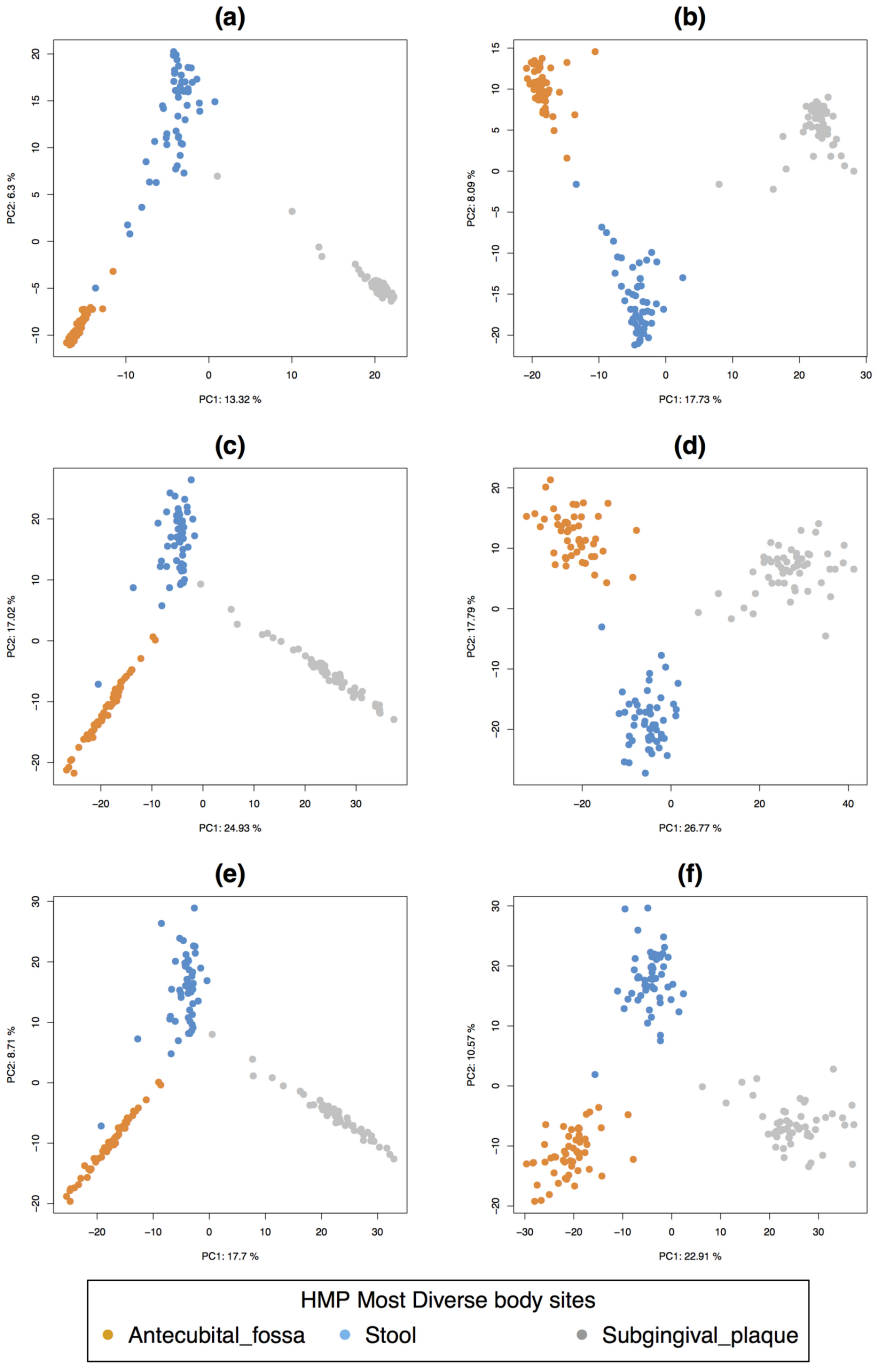
Most diverse data, PCA sample plots. **(a)** TSS and **(b)** TSS multilevel OTU log counts, **(c)** TSS-ILR and **(d)** TSS-ILR multilevel normalised log counts, **(e)** CSS and **(f)** CSS multilevel log counts.

We then compared the different normalisation strategies, including the multilevel variance decomposition using PCA. The normalisations TSS, TSS + ILR, CSS seemed to cluster the body sites similarly (Fig 3(a), 3(c) and 3(e)). The multilevel decomposition led to a smaller variability within body sites and a greater variability between body sites (Fig 3(b), 3(d) and 3(f)), and consequently increased the amount of variance explained. Using TSS+ILR or CSS also increased the explained variance (TSS+ILR, 44.6% for the first two components, 33.5% for CSS).

This preliminary exploration indicated that the abundance of microbial communities could characterise each body site quite clearly, and that the multilevel decomposition enabled better separation of the body site clusters, in particular when applied to the TSS+ILR or CSS normalised data.

### Supervised analysis on Most Diverse body sites dataset

We applied multilevel sPLS-DA to identify a microbiome signature characterising each body site and compared the different normalisation strategies (TSS+CLR or CSS) in our multivariate method to DESeq2 and ZIG univariate methods.

#### Impact of normalisation to identify discriminative features with sPLS-DA

**Fig 4:**
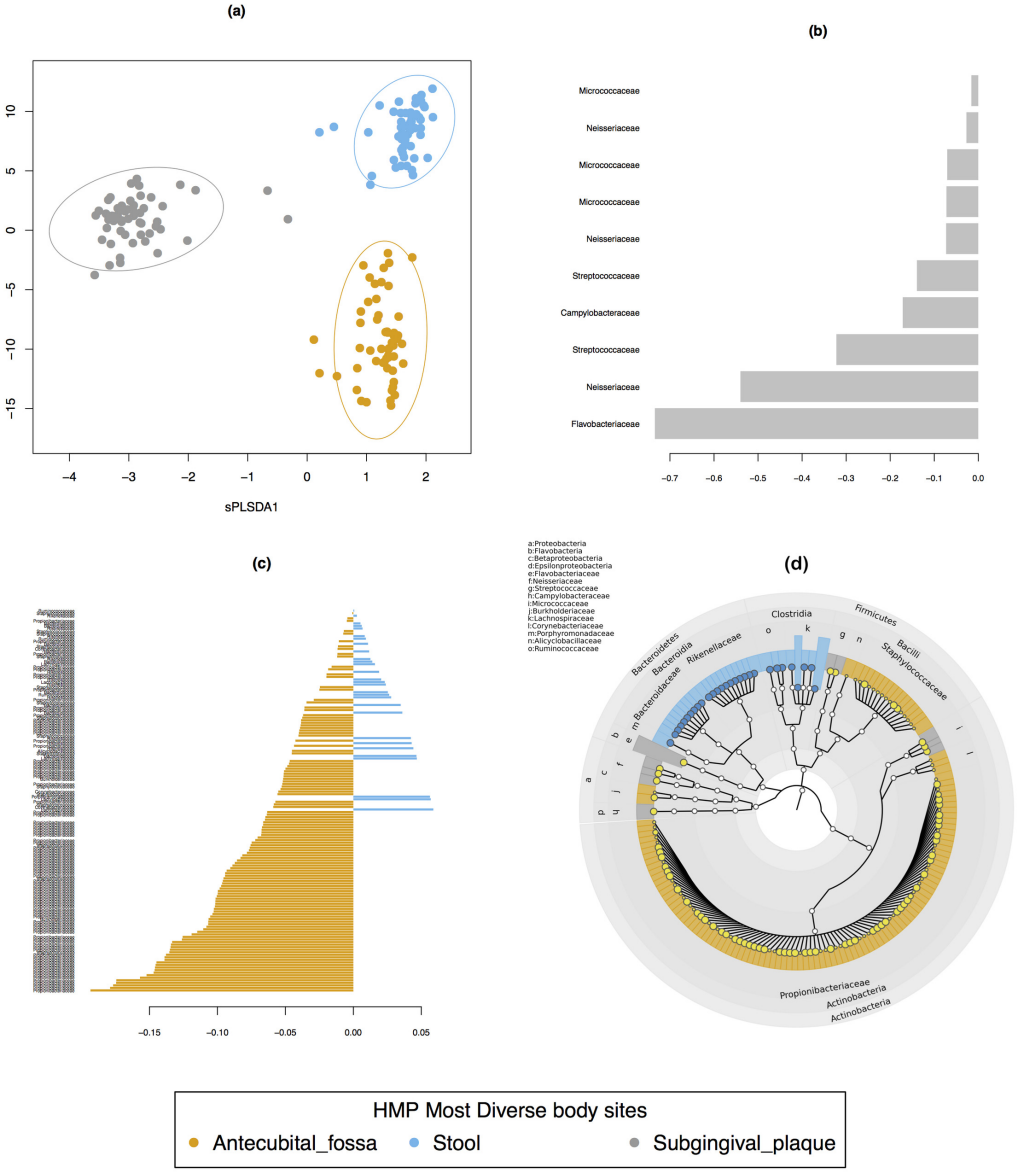
Most diverse TSS+CLR data, sPLS-DA sample, contribution and cladogram plots. **(a)** sample plot on the first two components with 95% confidence level ellipse plots, **(b)** and **(c)** represent the contribution of each OTU feature selected on the first (10 OTUs) and second component (120 OTUs), with OTU contribution ranked from bottom (important) to top. Colours indicate body site in which the OTU is most abundant. **(d)** Cladogram generated from the sPLS-DA result using GraphlAn.

The sPLS-DA classification performance was similar in both TSS+CLR or CSS normalised data. The lowest classification error rate was obtained for two components (0.7% for TSS+CLR and 0.3% for CSS, S4 Table). Both normalisations consistently misclassified antecubical fossa on the first component but correctly classified the two other body sites, and the addition of the second component enabled a better classification of all body sites (Fig 4). The number of OTUs selected with sPLS-DA was 160 with TSS+CLR and 130 with CSS. We next assessed the contribution of the selected OTU selected on each component (S5 Table). We found that both normalisations identified similar bacterial families. Component 1 characterised the subgingival plaque with *Micrococcaceae, Neisseriaceae, Streptococcaceae, Flavobacteriaceae* and *Campylobacteraceae*. CSS also identified the *Burkholderiaceae* family. Component 2 characterised stool and anticubital fossa. For anticubital fossa, TSS+CLR identified *Propionibacteriaceae, Staphylococcaceae and Corynebacteriaceae* while CSS additionally identified *Propionibacteriaceae, Staphylococcaceae* but failed to identify *Corynebacteriaceae*. Bacterial families characterising stool included *Bacteroides, Ruminococcaceae, Lachnospiraceae, Rikenellaceae* and *Porphyromonadaceae*. Across the three body sites, we found that both normalisations led to very similar families of bacteria—5 families for component 1, 10 (TSS+CLR) or 8 (CSS) for component 2 with a difference of 1 or 2 families on each component between TSS+CLR and CSS (see S5 Table). Interestingly, we observed that increasing the number of selected OTU did not add more relevant bacteria families. It is rather the proportion of number of OTU corresponding to the families that varied (Fig 4(d)).

#### Comparisons with no multilevel approach

To understand the impact and benefits of the proposed multilevel approach, we examined the OTU selected by sPLS-DA multilevel on either the TSS or CSS normalised counts without multilevel transformation. The classification error rate was substantially greater than with the previous multilevel analysis, (6% for TSS+CLR and 3% for CSS for two components) with a larger number of OTU selected (400 OTU selected for TSS+CLR and 240 for CSS). With the TSS+CLR normalisation, we identified similar families characterising subgingival plaque on the first component, including *Burkholderiaceae, Fusobacteriaceae, Gemellaceae, Veillonellaceae*. The families selected on the second component characterised antecubital fossa similarly to the multilevel approach, however the notable omission was the entire *Ruminococcus* family characterising stool in the multilevel approach that was not identified here. Overall, we found that the multivariate analysis ignoring the repeated-measures design tended to identify differential features driving the overall signature and disregarded subtleties between microbial communities in environments sampled on the same individuals.

#### Comparison with univariate analysis

While the number of OTUs declared as differentially abundant was similar between DESeq2 and ZIG (S2 Table), we observed strong differences at both OTU and family levels (S3 Fig). Interestingly, the sPLS-DA selections were all included in the ZIG and DESEq2 selections. DESeq2 identified relevant features that were common to sPLS-DA selections, such as *Propionibacteriaceae, Staphylococcaceae* and *Corynebacteriaceae* with the addition of *Burkholderiaceae* as a defining feature characterising Antecubital fossa. It also characterised the Subgingival plaque microbial community with OTUs from *Streptococcaceae, Neisseriaceae, Gemellaceae* and *Micrococcaceae* families, also identified in sPLS-DA. However, DESeq2 was poor at characterising Stool. Indeed, very few bacterial families, including *Bacteroides* and *Lachnospiraceae* were identified. Such low bacterial diversity was not consistent with the sPLS-DA nor with the literature. Similar to DESeq2 and sPLS-DA, ZIG identified features of the Antecubital fossa with OTU belonging to *Propionibacteriaceae, Staphylococcaceae, Burkholderiaceae* and *Corynebacteriaceae*. Like DESeq2, ZIG described the Subgingival plaque microbiome with OTU belonging to *Streptococcaceae, Neisseriaceae, Micrococcaceae* and *Gemellaceae*. However, ZIG also identified OTUs belonging to *Fusobacteriaceae, Burkholderiaceae, Flavobacteriaceae, Campylobacteraceae, Veillonellaceae* and *Actinomycetaceae*. In contrast to DESeq2, ZIG identified and described the Stool microbiome well, with OTU belonging to the families of *Bacteroides, Porphyromonadaceae, Rikenellaceae, Lachnospiraceae* and *Ruminococcaceae*. One reason to explain the differences between the two univariate methods might be that DESeq2 does not adequately model sparse counts.

### Analysis of the oral body site dataset with mixMC

Similar to the Most Diverse data set, unsupervised data analyses showed that unweighted Unifrac better discriminated the different body sites (plaque, gingiva) compared to weighted Unifrac in the PCoA sample plots (S4(a) and S4(b) Fig). TSS+ILR explained greater variance (21.35% on the first component) than CSS (13.63%), with better separated body sites clusters (S4(c) and S4(e) Fig). The explained variance further increased with a multilevel variance decomposition (25.37% vs. 18.22%, S4(d) and S4(f) Fig).

#### sPLS-DA performance and choice of parameters

We observed similar classification performances between sPLS-DA on either TSS+CLR or CSS, with a slightly lower classification error rate for TSS+CLR (S5 Fig, Table 1). The final sPLS-DA model included 8 components that led to optimal performance, with a classification error rate that substantially decreased from 78% (component 1) to 26% for TSS+CLR and 30% for CSS (component 8). The classification error rate remained relatively high as similar body sites were consistently misclassified across components, as described in Table 1. For example, Tonsils had the highest classification error rate as no OTU was able to characterise this particular body site (Table 1). We observed that the TSS+CLR normalisation was better at characterising tonsil and plaque (component 1), buccal mucosa (component 2) and gingiva (component 3) than the CSS normalisation. The CSS normalisation also led to a substantial number of ties (equal median counts) when assessing the body site contribution of the selected OTU (not shown). Therefore, the detailed analysis that follows solely focuses on a multilevel sPLS-DA model with TSS+CLR normalisation.

**Table 1:** Oral data. Top: Number of selected features at the OTU (family) level and mean classification error rate per component. Bottom: Number of features at the OTU (family) level contributing to each body site for each sPLS-DA component. Note that we may observe some overlap between families across the different body sites.

	Comp 1	Comp 2	Comp 3	Comp 4	Comp 5	Comp 6	Comp 7	Comp 8
# features selected	60 (13)	40 (2)	190 (18)	200 (14)	40 (8)	200 (26)	180 (23)	190 (22)
mean classification error rate	0.778	0.584	0.501	0.410	0.336	0.316	0.279	0.262
sd classification error rate	0.000	0.002	0.003	0.003	0.003	0.005	0.004	0.004
Attached Keratinized gingiva	0	35 (2)	123 (12)	9 (6)	1 (1)	73 (16)	34 (11)	47 (15)
Buccal mucosa	0	5 (1)	4 (1)	1 (1)	0	31 (4)	3 (1)	3 (1)
Hard palate	2 (1)	0	1 (1)	3 (1)	0	3 (2)	5 (3)	9 (3)
Palatine Tonsils	1 (1)	0	0	5 (3)	0	2 (2)	4 (2)	6 (4)
Saliva	5 (3)	0	2 (2)	28 (5)	0	4 (2)	11 (5)	7 (2)
Subgingival plaque	0	0	7 (7)	15 (5)	39 (7)	14 (11)	6 (5)	21 (10)
Supragingival plaque	11 (4)	0	53 (8)	23 (6)	0	31 (9)	15 (8)	31 (6)
Throat	11 (5)	0	0	16 (4)	0	5 (3)	42 (5)	9 (4)
Tongue dorsum	30 (9)	0	0	100 (8)	0	37 (11)	60 (13)	57 (12)

#### Body sites characterisation

**Fig 5:**
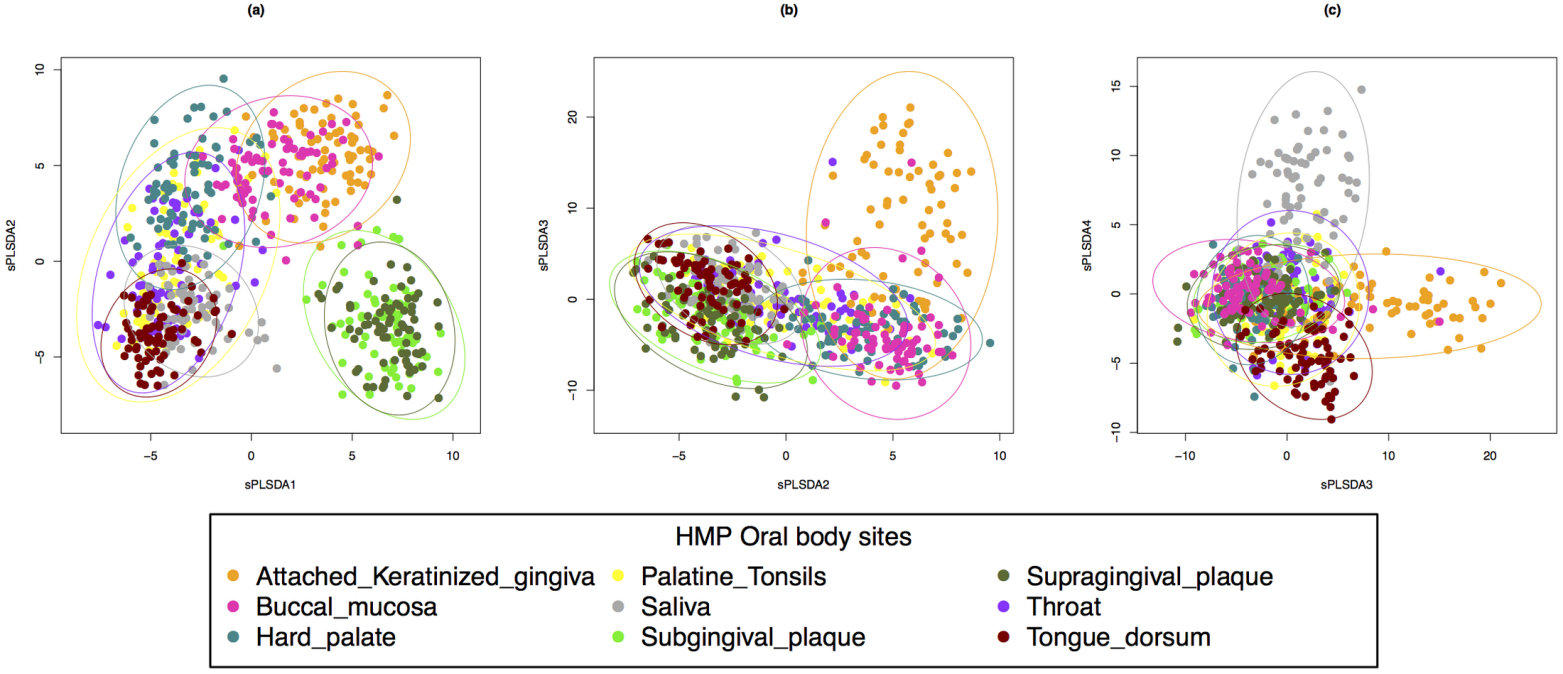
Oral data, sPLS-DA sample plot for the different components. **(a)** Component 1 vs. Component 2, **(b)** Component 2 vs Component 3, using 95% confidence ellipses.

We mainly focused on the first three sPLS-DA components for our interpretation (Fig 5 and S6 Fig for the remaining 5 components). Each component seemed to characterise specific subsets of the body sites. For example component 1 discriminated sub and supra gingival plaque against the other body sites, component 2 clustered attached keratinised gingiva and buccal mucosa, but with no clear cut separation (Fig 5(a)), while component 3 seemed to separate attached keratinised gingiva form the others (Fig 5(c)). Similar conclusions could be drawn for the other components (S6 Fig). The interpretation of these sample plots can be subjective, however, they reflect the close anatomical proximity of the different sample sites in the mouth, such as the tongue coming in contact with the hard palate, teeth, saliva and gums.

#### Features contribution

**Fig 6:**
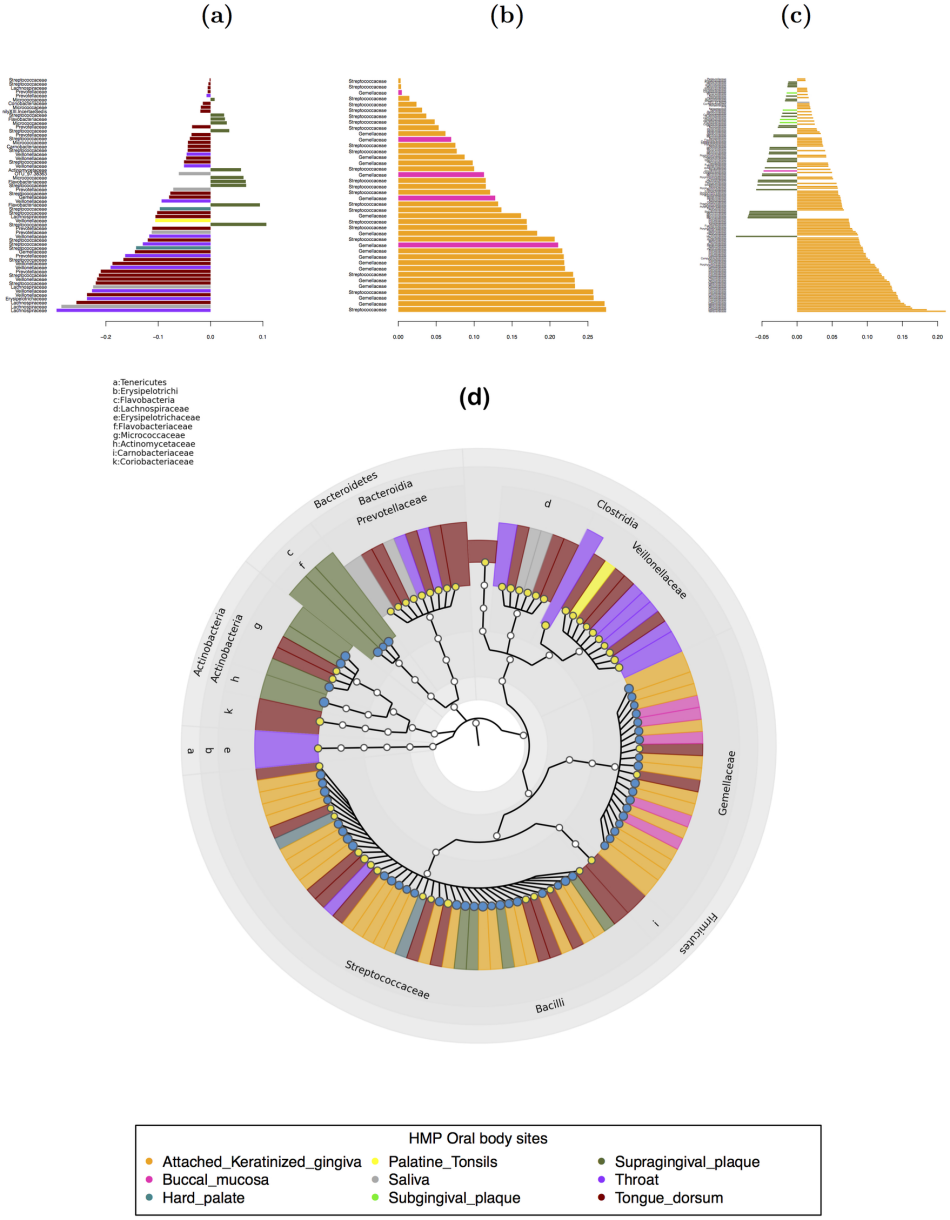
Oral data, contribution and cladogram plots of the features selected for each sPLS-DA component. **(a)** Component 1, **(b)** Component 2, **(c)** Component 3. In **(c)** only the top 150 OTU are represented. **(d)** Cladogram generated from the sPLS-DA results for components 1 and 2 using GraphlAn.

Table 1 details the number of features contributing to each oral site per component. Those outputs combined with the interpretation from the sample plots in Fig 5 enable better insight into bacteria contributing to body sites that are contiguous. For some cases we observed similar contributions of microbial communities in close body sites, for example Throat and Tongue appeared to be characterised by the same family of bacteria. The closeness of those selected bacteria in terms of their taxonomy can be visualised in the cladogram in Fig 6(d). We examined the ability of sPLS-DA to highlight subtle differences and characterise different sites in close proximity within the oral microbiome. We reviewed the relevant families selected on the first three sPLS-DA components, which appeared to characterise particular body sites (Table 1, S1 File).

The bacteria families selected on component 1 strongly characterised hard palate (members of the *Streptococcaceae* family), saliva (*Prevotellaceae*, *Lachnospiraceae* as well as the phylum TM7 recently described in [48] and found prevalent in oral cavity), supragingival plaque as well as throat and tongue. The throat microbiome was characterised by *Prevotellaceae, Lachnospiraceae, Veillonellaceae, Streptococcaceae* and *Erysipelotrichaceae*. The tongue was found to be more diverse with eight families of bacteria found to be characterising the site. These include the order *Clostridiales* families *Coriobacteriaceae, Gemellaceae, Carnobacteriaceae, Lachnospiraceae, Prevotellaceae, Micrococcaceae, Streptococcaceae* and *Veillonellaceae*. Component 2 separated attached keratinized gingiva from buccal mucosa with the families *Gemellaceae* and *Streptococcaceae*. Component 3 discriminated multiple sites, in particular attached keratinized gingiva (*Prevotellaceae, Porphyromonadaceae, Flavobacteriaceae, Carnobacteriaceae, Streptococcaceae, Fusobacteriaceae, Campylobacteraceae, Pasteurellaceae, Neisseriaceae, Moraxellaceae* and TM7), buccal mucosa and hard palate (*Streptococcaceae* for both). Interestingly, component 3 discriminated subgingival plaque (*Burkholderiaceae, Flavobacteriaceae, Gemellaceae, Micrococcaceae, Neisseriaceae, Prevotellaceae* and *Streptococcaceae*) from supragingival plaque (*Actinomycetaceae, Burkholderiaceae, Flavobacteriaceae, Fusobacteriaceae, Micrococcaceae, Neisseriaceae* and *Streptococcaceae*) with some overlap between the families.

The analysis of the Oral dataset using our mixMC framework identified relevant bacteria families characterising subtle differences in the oral environment as well as deciphering particular characteristics in each body site.

### Comparison with the Koren data set

To further validate the relevance of our multivariate method to discriminate and identify microbial features describing microbial communities, we applied our sPLS-DA to the study from Koren *et al*. [25]. Since the dataset only contained partially repeated measures from multiple sites (individual patients samples in plaque were not sampled in other body sites), we applied a non multilevel sPLS-DA on the TSS+CLR data, resulting in a selection of 30+100 OTU on two components (S7 Fig, S1 File). We found that sPLS-DA was able to clearly and distinctly discriminate the three body sites saliva, plaque and stool. Component 1 best characterised stool identifying families of bacteria such as *Lachnospiraceae, Ruminococcaceae and Bacteroides*; similar to what was observed in the HMP dataset. Component 2 best discriminated arterial plaque and saliva. Arterial plaque was characterised by families including *Burkholderiaceae, Propionibacteriaceae, Pseudomonadaceae and Staphylococcaceae*, which was consistent with what the authors reported to as the ‘core microbiome’ for arterial plaque samples. Our analysis also identified *Alcaligenaceae, Enterobacteriaceae, Moraxellaceae* and *Comamonadaceae* as bacterial families describing arterial plaque. Saliva was also characterised on component 2 by the same families of bacteria both reported by Koren *et al*. and our microbiome signature in the HMP data set.

Our comparative analysis demonstrates that sPLS-DA not only produced reliable and consistent results across different sequencing platforms and datasets but was also able to identify key members of the microbial community characterising in particular saliva, plaque and stool.

## Discussion

Traditionally, unsupervised dimension reduction multivariate approaches for microbiome data such as PCoA use pairwise distances or dissimilarities calculated on count data to scale microbial community abundances. However, the output of such method is limited to the visualisation of patterns in the data only. Our mixMC framework did not propose such distances for various reasons. From a theoretical point of view and as discussed by Warton *et al*. [49], distance-based analyses make implicit assumptions on the mean-variance relationship in count data that may not hold, with the consequence of possible misleading results. From a practical point of view, a multivariate projection based method applied on a *n* × *n* similarity matrix does not enable identification of bacteria driving differences between habitats. We therefore proposed to directly handle abundance data to achieve that goal.

In our study, we compared two normalisation techniques for 16S OTU count data. TSS normalisation is a popular approach to accommodate for varying sampling and sequencing depth [8, 22], but with the disadvantage of producing compositional data that may lead to spurious results when applying traditional statistical methods [13, 15]. Transforming compositional data using log ratios such as Isometric Log Ratio (ILR) or Centered Log Ratio transformation (CLR) enables to circumvent this issue [10, 32]. Our mixMC framework includes those transformations to visualise diversity patterns (PCA) or to perform discriminant analysis and identify indicator species explaining abundance differences between habitats (sPLS-DA). We applied the ILR transformation for PCA, as proposed by [16, 32] to overcome the CLR limitation that may lead to singular covariance matrices. For sPLS-DA however, the feature selection process requires *n* × *p* input matrix in order to identify indicator species and we therefore applied the one-to-one CLR transformation. We showed that sPLS-DA delivered relevant results in our three case studies using TSS+CLR transformed data. CSS normalisation was proposed by Paulson *et al*. to account for sparse counts [9]. In the Most Diverse case study we showed that both TSS and CSS normalisations identified the same bacteria families. In the more complex Oral case study we observed differences as TSS+CLR led to the identification of a greater number of families than CSS. We therefore must therefore keep in mind that normalisation is data specific and needs to be carefully chosen prior to statistical analysis.

Our mixMC framework proposes to handle repeated-measures design with a multilevel variance decomposition. This additional transformation step can also be seen as a scaling transformation to be able to extract subtle differences between body sites or habitats within the same individuals. We anticipate that such experimental designs will become widely adopted in microbiome studies. However, our framework is not only restricted to repeated measures designs and can be used in a more general case to compare phenotypes or disease outcomes.

mixMC proposes more extensive analytical features than univariate methods, including insightful graphical outputs for data interpretation. We found that both univariate and multivariate approaches led to similar overall structure of the signatures were similar at the family level. However, dimension reduction multivariate approaches provide intuitive plots and numerical outputs for a better understanding of the discriminative ability of the OTU features identified.

Our study aligns well with recent studies that investigated the link between gut and oral microbial communities [25, 50]. Franzosa *et al*. showed identified a subset of abundant oral microbes surviving transit to the gut that were linked with disease markers of atherosclerosis such as cholesterol [50]. From our detailed analyses, we reached similar conclusions identifying bacteria such as *Fusobacterium*, *Propionibacterium, Veillonella* in both the oral body sites from both HMP data sets (including plaque, tongue and gingiva) and stool microbiomes as underlined by Koren *et al*. [25]. Our comparative study with the Koren data set demonstrated that sPLS-DA was able to identify a microbiome signature consistent across different individual cohorts and sequencing platforms. The microbiome signatures we identified from the most diverse HMP data set and the Koren data set further demonstrated that microbial communities can not be considered discrete environments, but are, in fact, fluid environments.

## Conclusions

mixMC is a statistical analysis framework enabling holistic understanding of microbial communities. In this study, we demonstrated the advantages of using multivariate methodologies for the statistical analysis of 16S compositional data, to summarise and reduce the dimension of possibly large data sets; to obtain a better understanding of the microbial communities through insightful graphical outputs; and to highlight features characterising and discriminating different environments. While our study has particularly focused on repeated-measures designs, the multivariate approach that we propose is not restricted to such designs only. Similar analyses can be performed on non-repeated designs to highlight relevant microbial features.

The multivariate approach sPLS-DA is a specific case of a larger family of projection-based multivariate approaches, some of which also allow integration of different types of data. Our proposed analysis framework therefore paves the transition towards a ‘microbiome system biology’ approach by integrating large scale multi-‘omics studies such as metatranscriptomics, metabolomics or metaproteomics currently being collected by the integrative HMP project [51], therefore enabling the improvement of our understanding of the biomolecular activities and regulatory systems of human microbiota.

### Availability of supporting data

The data sets supporting the results of this article are available from the NIH Human Microbiome Project http://hmpdacc.org/HMQCP/all/ in raw data format, and in processed format on our website www.mixOmics.org/mixMC. R functions are available on our mixOmics package [41, 52]. R scripts and a full tutorial to reproduce the results from the proposed framework are also available on our website.

## Supporting Information

S1 Text Isometric Log Ratio transformation. (PDF)

S1 Table Description of the two HMP data sets through preprocessing steps. (PDF)

S2 Table Most diverse data, number of features selected by the different univariate and multivariate approaches at the OTU or family level. The OTU selection is based on either 5% significance level (adjusted FDR p-values) for DESeq2 and ZIG or the best classification performance with mean error rate across 10-fold cross-validation repeated 100 times (standard deviation) for sPLS-DA with two components.
(PDF)

S3 Table Oral data, performance of sPLS-DA per component and body site (TSS+CLR data). The mean classification error rate across 10-fold cross validation performed 100 times is indicated.
(PDF)

S4 Table Most diverse data, performance of sPLS-DA per body site. Componentwise 100*10-fold cross-validation classification error rate for sPLS-DA applied to either TSS+CLR or CSS normalised counts with respect to each body site class leading to the optimal microbiome signature.
(PDF)

S5 Table Most diverse data, number of features contributing to each body site for each sPLS-DA component. The sPLS-DA model was applied to either TSS+CLR or CSS normalised counts. Contribution is defined as the body site for which the maximum median normalised OTU abundance is achieved at the OTU (family) level.
(PDF)

S1 Fig Oral data, PCoA sample plots with colours indicating gender or run centres. Sample plot on the first two coordinates with colours indicating gender in **(a)** weighted Unifrac or **(b)** unweighted Unifrac, or run centers in **(c)** weighted Unifrac or **(d)** unweighted Unifrac calculated on the filtered OTU count table.
(TIF)

S2 Fig Most diverse data, PCoA sample plots. Sample plot on the first two coordinates with **(a)** weighted Unifrac **(b)** unweighted Unifrac calculated on the unfiltered OTU count table (based on 43,146 OTU).
(TIF)

S3 Fig Most diverse data, comparison between univariate OTU selections and multivariate sPLS-DA selection. Comparison of the most differentially abundant features identified by DESeq2 and ZIG (FDR ≤ 0.05) and the most discriminative features identified by TSS+CLR with sPLS-DA or CSS withsPLS-DA (lowest mean classification error rate achieved when performing 100 * 10-fold cross-validation). **(a)**: selection size at OTU level, **(b)**: at the family level.
(TIF)

S4 Fig Oral data, PCoA and PCA sample plots. Sample plot on the first two coordinates with **(a)** weighted Unifrac **(b)** unweighted Unifrac calculated on the filtered OTU count table and on the first components for **(c)** TSS+ILR and **(d)** TSS+ILR multilevel normalised OTU counts, and **(e)** CSS and **(f)** CSS multilevel normalised OTU counts.
(TIF)

S5 Fig Oral data, sPLS-DA performance. Mean classification performance using 100 * 10-fold cross-validation. Each component is based on an optimal selection of OTU features that leads to the best classification performance. The sPLS-DA classifier was applied on **(a)** TSS+CLR or **(b)** CSS normalised data.
(TIF)

S6 Fig Oral data, sPLS-DA sample representation for the different components of the model. **(d)** Component 4 vs Component 5, **(e)** Component 5 vs Component 6, **(f)** Component 6 vs Component 7, **(g)** Component 7 vs Component 8.
(TIF)

S7 Fig Koren data. Sample plot on the first two components with **(a)** PCA **(b)** sPLS-DA on selected OTU. Contribution plots on the **(c)** first component (30 OTU selected) and **(d)** on the second component (100 OTU selected).
(TIF)

S1 File Diverse, Oral and Koren TSS+CLR data: selected OTU. Contribution of selected OTU for each sPLS-DA component.
(ZIP)
