# Supplementary material for: MixMC: A Multivariate Statistical Framework to Gain Insight into Microbial Communities
Source: PLoS One. 2016 Aug 11;11(8):e0160169. doi: 10.1371/journal.pone.0160169 (PMC4981383; doi:10.1371/journal.pone.0160169)
Supplement: S1 Text — (PDF) [file pone.0160169.s001.pdf]

Supporting Information

S1 Material

Isometric Log Ratio transformation.

Isometric Log Ratio transformation. ILR transformed data $(z_1, \ldots, z_{p-1})'$ are spanned by $(p-1)$ new coordinates with respect to the orthonormal basis $\boldsymbol{V}$. Each composition x_i ($i = 1, \ldots, p$) is ILR transformed as:

$$z_i = v_i * log \frac{\sqrt[i]{\prod_{j=1}^{i} x_j}}{x_{i+1}} \sqrt{\frac{p-i}{p-i+1}} * y_i v_i = \sqrt{\frac{p-i}{p-i+1}}$$

with the orthonormal basis vector $v_i = \sqrt{\frac{i}{i+1}}$ $(i = 1, \ldots, p-1)$ [2]. Note that ILR transformed data are not easily interpretable as they are of dimension $p-1$ (i.e. there is not a one-to-one transformation of all features). Therefore, Filmoser *et al.* proposed to back transform the PCA results to the CLR space using the linear relationship between CLR and ILR transformations [1]: $\boldsymbol{y} = \boldsymbol{V}\boldsymbol{z}$, where $\boldsymbol{V} = (v_1, \ldots, v_{p-1})$ is a $p \times (p-1)$ matrix with orthonormal basis vectors v_i as defined above. The ILR followed by back transformation was used in `mixMC` with PCA.

References

[1] Filzmoser, P., Hron, K., Reimann, C.: Principal component analysis for compositional data with outliers. Environmetrics 20(6), 621-632 (2009)

[2] Egozcue, J.J., Pawlowsky-Glahn, V., Mateu-Figueras, G., Barcelo-Vidal, C.: Isometric logratio transformations for compositional data analysis. Mathematical Geology 35(3), 279-300 (2003)
