# Supplementary material for: MixMC: A Multivariate Statistical Framework to Gain Insight into Microbial Communities
Source: PLoS One. 2016 Aug 11;11(8):e0160169. doi: 10.1371/journal.pone.0160169 (PMC4981383; doi:10.1371/journal.pone.0160169)
Supplement: S1 Table — (PDF) [file pone.0160169.s002.pdf]

Supporting Information

S1 Table

Table S1: Description of the two HMP data sets through preprocessing steps.

	Most Diverse body sites	Oral body sites
# initial OTU	43,140	43,140
# raw OTU after pre-filtering	1,674	1,562
# samples	162	657
# unique individuals	54	73
# body sites	3	9
