# Supplementary material for: MixMC: A Multivariate Statistical Framework to Gain Insight into Microbial Communities
Source: PLoS One. 2016 Aug 11;11(8):e0160169. doi: 10.1371/journal.pone.0160169 (PMC4981383; doi:10.1371/journal.pone.0160169)
Supplement: S2 Table — The OTU selection is based on either 5% significance level (adjusted FDR p-values) for DESeq2 and ZIG or the best classification performance with mean error rate across 10-fold cross-validation repeated 100 times (standard deviation) for sPLS-DA with two components. (PDF) [file pone.0160169.s003.pdf]

Supporting Information

S2 Table

Table S2: **Most diverse data, number of features selected by the different univariate and multivariate approaches at the OTU or family level.** The OTU selection is based on either 5% significance level (adjusted FDR p-values) for DESeq2 and ZIG or the best classification performance with mean error rate across 10-fold cross-validation repeated 100 times (standard deviation) for sPLS-DA with two components.

**Feature selection method**	DESeq2	ZIG	TSS+CLR with sPLS-DA	CSS with sPLS-DA
**# OTU level**	883	742	160	130
**# family level**	39	34	15	14
**Criterion**	adj. p-val $\leq$ 0.05	adj. p-val $\leq$ 0.05	mean error: 0.007 (sd: 0.003)	mean error: 0.003 (sd: 0.005)
