# Supplementary material for: MixMC: A Multivariate Statistical Framework to Gain Insight into Microbial Communities
Source: PLoS One. 2016 Aug 11;11(8):e0160169. doi: 10.1371/journal.pone.0160169 (PMC4981383; doi:10.1371/journal.pone.0160169)
Supplement: S3 Table — The mean classification error rate across 10-fold cross validation performed 100 times is indicated. (PDF) [file pone.0160169.s004.pdf]

Supporting Information

S3 Table

Table S3: **Oral data, performance of sPLS-DA per component and body site (TSS+CLR data).** The mean classification error rate across 10-fold cross validation performed 100 times is indicated.

	comp 1	comp 2	comp 3	comp 4	comp 5	comp 6	comp 7	comp 8
Attached Keratinized Gingiva	1.00000	0.02603	0.09041	0.10164	0.10630	0.10795	0.11205	0.08411
Buccal Mucosa	1.00000	1.00000	0.19151	0.19973	0.21753	0.24685	0.23397	0.27151
Hard Palate	1.00000	0.24795	0.21397	0.32767	0.34740	0.10877	0.15370	0.14301
Palatine Tonsils	1.00000	1.00000	1.00000	1.00000	0.70959	0.70356	0.73890	0.53315
Saliva	1.00000	1.00000	1.00000	0.04493	0.04712	0.06137	0.06877	0.08603
Subgingival Plaque	1.00000	1.00000	0.99836	0.99123	0.38740	0.38466	0.39260	0.39288
Supragingival Plaque	0.00000	0.01370	0.01507	0.01945	0.20301	0.20384	0.19836	0.19315
Throat	1.00000	0.97425	0.99562	0.99644	0.99973	0.99808	0.53671	0.54630
Tongue Dorsum	0.00000	0.00055	0.00712	0.00000	0.01397	0.01425	0.09205	0.08904
