# Supplementary material for: MixMC: A Multivariate Statistical Framework to Gain Insight into Microbial Communities
Source: PLoS One. 2016 Aug 11;11(8):e0160169. doi: 10.1371/journal.pone.0160169 (PMC4981383; doi:10.1371/journal.pone.0160169)
Supplement: S4 Table — Componentwise 100*10-fold cross-validation classification error rate for sPLS-DA applied to either TSS+CLR or CSS normalised counts with respect to each body site class leading to the optimal microbiome signature. (PDF) [file pone.0160169.s005.pdf]

Supporting Information

S4 Table

Table S4: **Most diverse data, performance of sPLS-DA per body site.** Componentwise 100*10-fold cross-validation classification error rate for sPLS-DA applied to either TSS+CLR or CSS normalised counts with respect to each body site class leading to the optimal microbiome signature.

Normalisation	Component	Antecubital Fossa	Stool	Subgingival Plaque	Overall
TSS+CLR	1	1.000	0.000	0.000	0.335 (0.000)
	2	0.000	0.000	0.020	0.007 (0.004)
	3	0.000	0.000	0.015	0.005 (0.004)
CSS	1	0.996	0.004	0.004	0.335 (0.008)
	2	0.002	0.000	0.008	0.003 (0.005)
	3	0.000	0.000	0.023	0.008 (0.003)
