# Supplementary material for: MixMC: A Multivariate Statistical Framework to Gain Insight into Microbial Communities
Source: PLoS One. 2016 Aug 11;11(8):e0160169. doi: 10.1371/journal.pone.0160169 (PMC4981383; doi:10.1371/journal.pone.0160169)
Supplement: S5 Table — The sPLS-DA model was applied to either TSS+CLR or CSS normalised counts. Contribution is defined as the body site for which the maximum median normalised OTU abundance is achieved at the OTU (family) level. (PDF) [file pone.0160169.s006.pdf]

Supporting Information

S5 Table

Table S5: **Most diverse data, number of features contributing to each body site for each sPLS-DA component.** The sPLS-DA model was applied to either TSS+CLR or CSS normalised counts. Contribution is defined as the body site for which the maximum median normalised OTU abundance is achieved at the OTU (family) level.

Normalisation	Component	Antecubital Fossa	Stool	Subgingival Plaque	Total
TSS+CLR	1	0	0	10 (5)	10 (5)
	2	121 (5)	29 (5)	0	150 (10)
CSS	1	0	0	10 (6)	10 (6)
	2	60 (3)	60 (5)	0	120 (8)
