# Supplementary figures and images for: MixMC: A Multivariate Statistical Framework to Gain Insight into Microbial Communities
Source: PLoS One. 2016 Aug 11;11(8):e0160169. doi: 10.1371/journal.pone.0160169 (PMC4981383; doi:10.1371/journal.pone.0160169)

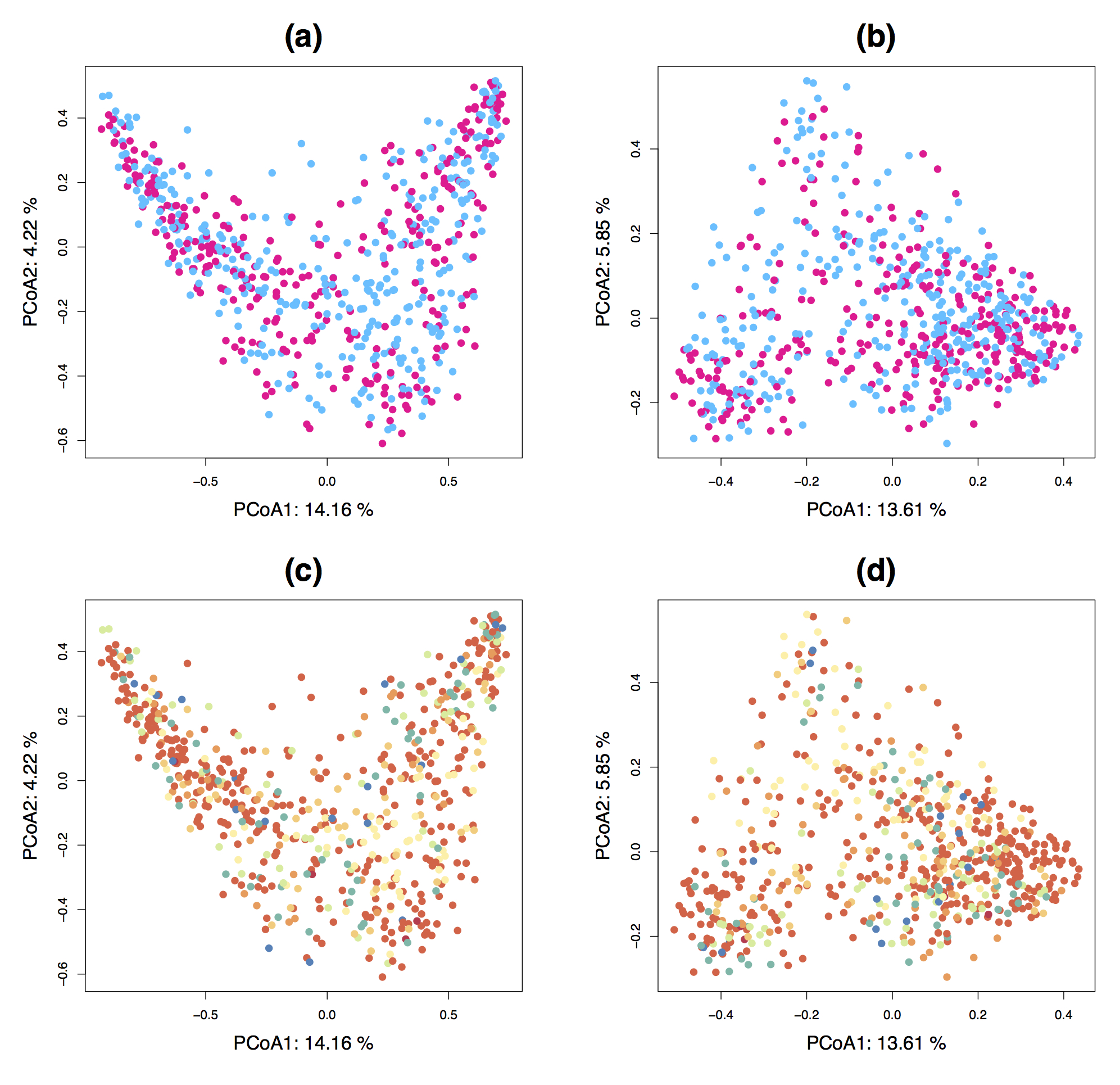

Supplement: S1 Fig — Sample plot on the first two coordinates with colours indicating gender in (a) weighted Unifrac or (b) unweighted Unifrac, or run centers in (c) weighted Unifrac or (d) unweighted Unifrac calculated on the filtered OTU count table. (TIF) [file pone.0160169.s007.tif]

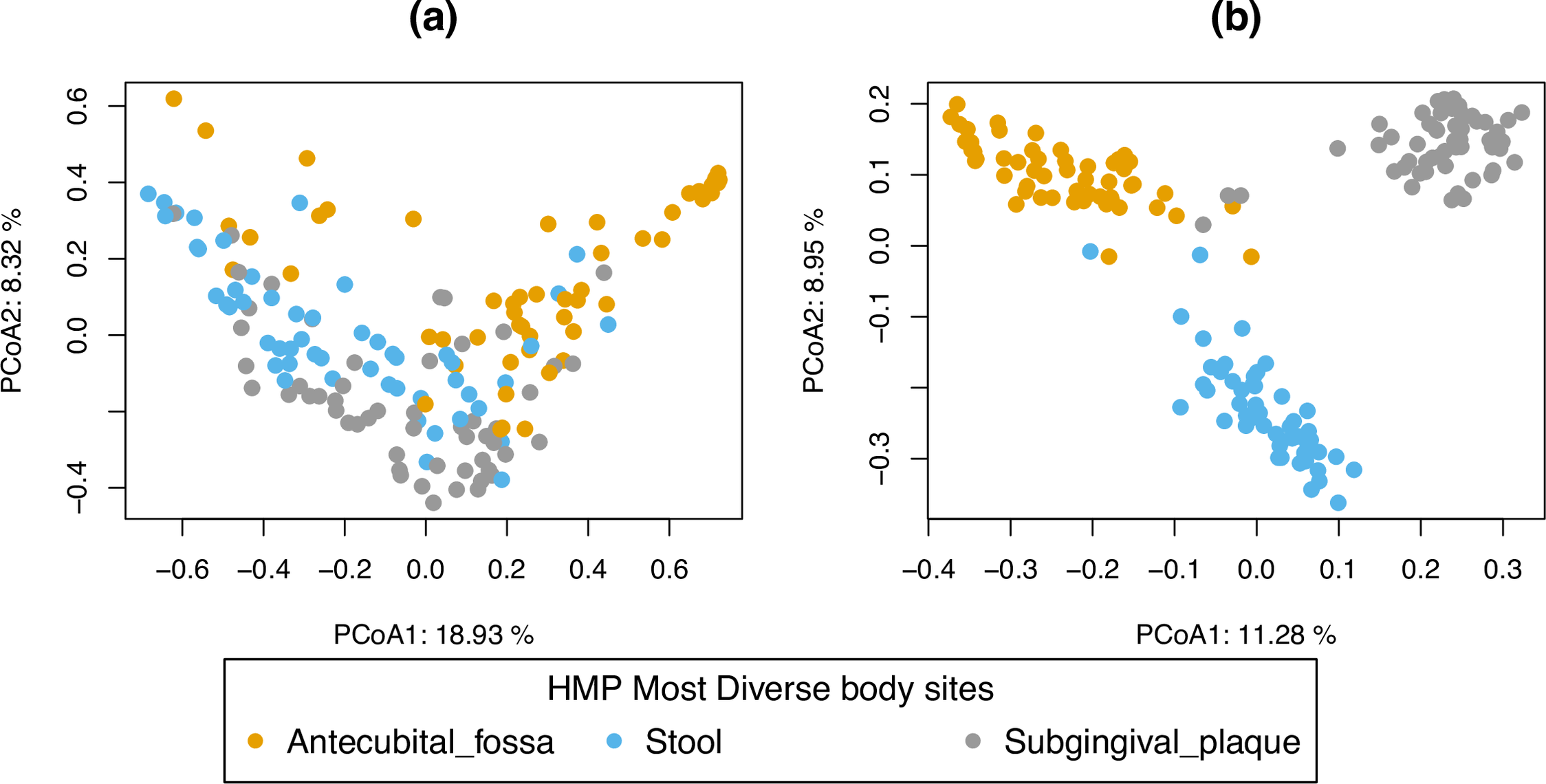

Supplement: S2 Fig — Sample plot on the first two coordinates with (a) weighted Unifrac (b) unweighted Unifrac calculated on the unfiltered OTU count table (based on 43,146 OTU). (TIF) [file pone.0160169.s008.tif]

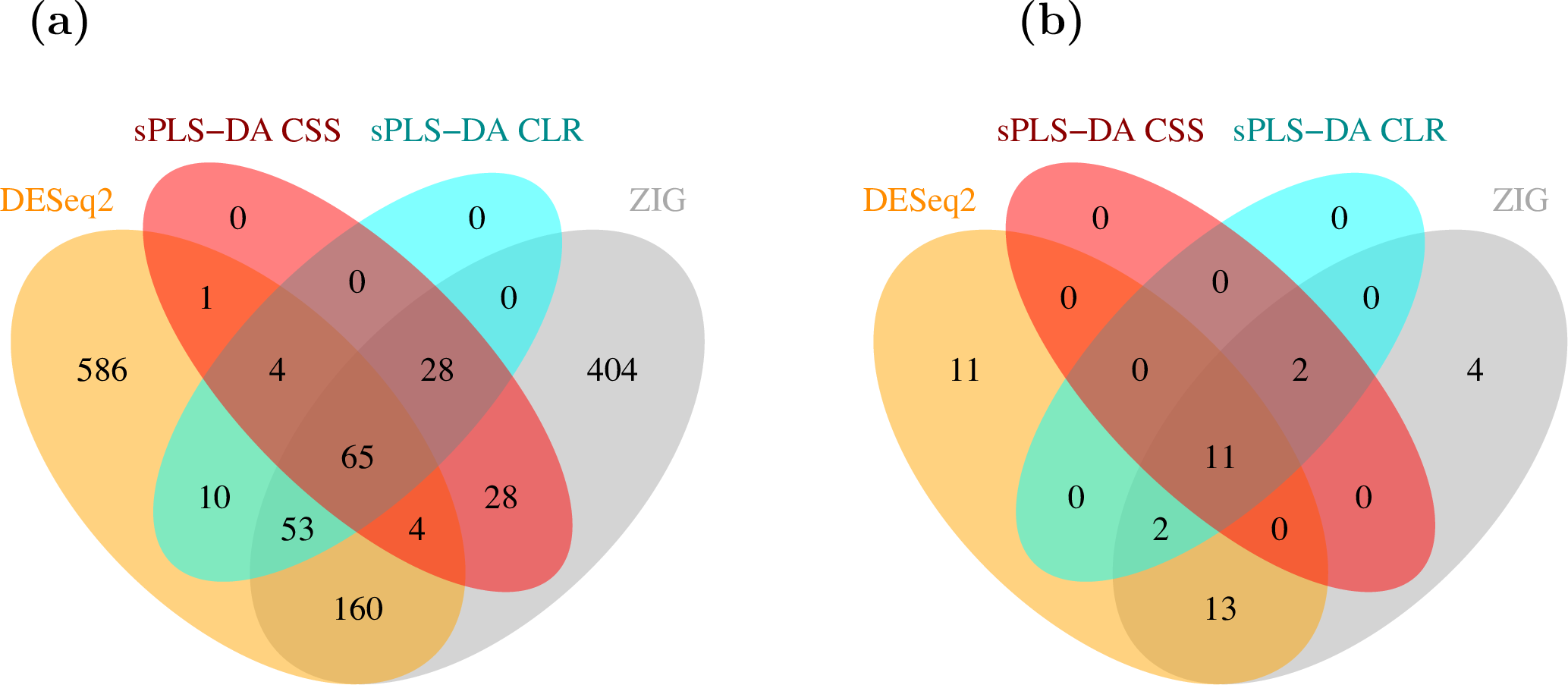

Supplement: S3 Fig — Comparison of the most differentially abundant features identified by DESeq2 and ZIG (FDR ≤ 0.05) and the most discriminative features identified by TSS+CLR with sPLS-DA or CSS withsPLS-DA (lowest mean classification error rate achieved when performing 100 * 10-fold cross-validation). (a): selection size at OTU level, (b): at the family level. (TIF) [file pone.0160169.s009.tif]

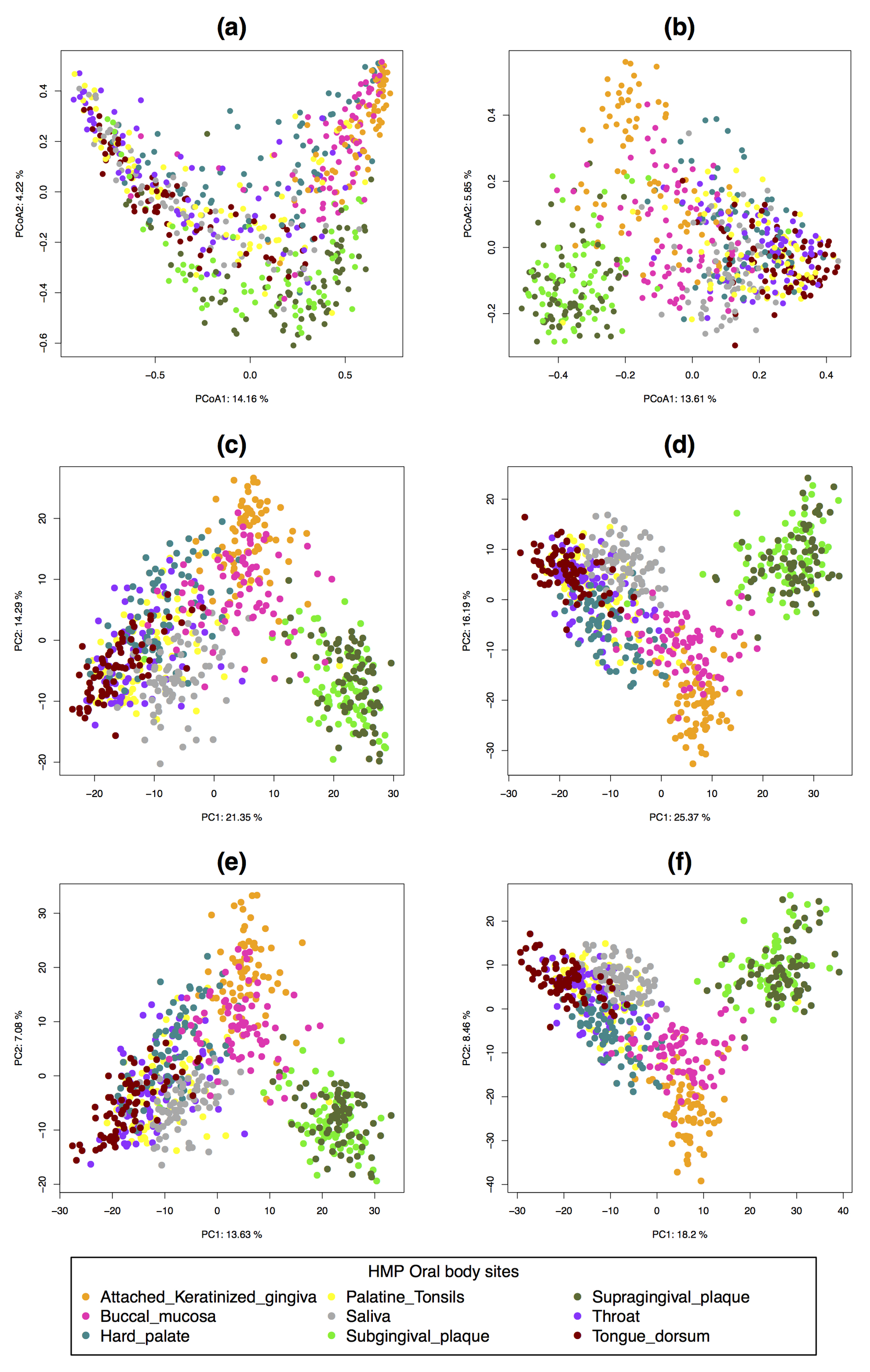

Supplement: S4 Fig — Sample plot on the first two coordinates with (a) weighted Unifrac (b) unweighted Unifrac calculated on the filtered OTU count table and on the first components for (c) TSS+ILR and (d) TSS+ILR multilevel normalised OTU counts, and (e) CSS and (f) CSS multilevel normalised OTU counts. (TIF) [file pone.0160169.s010.tif]

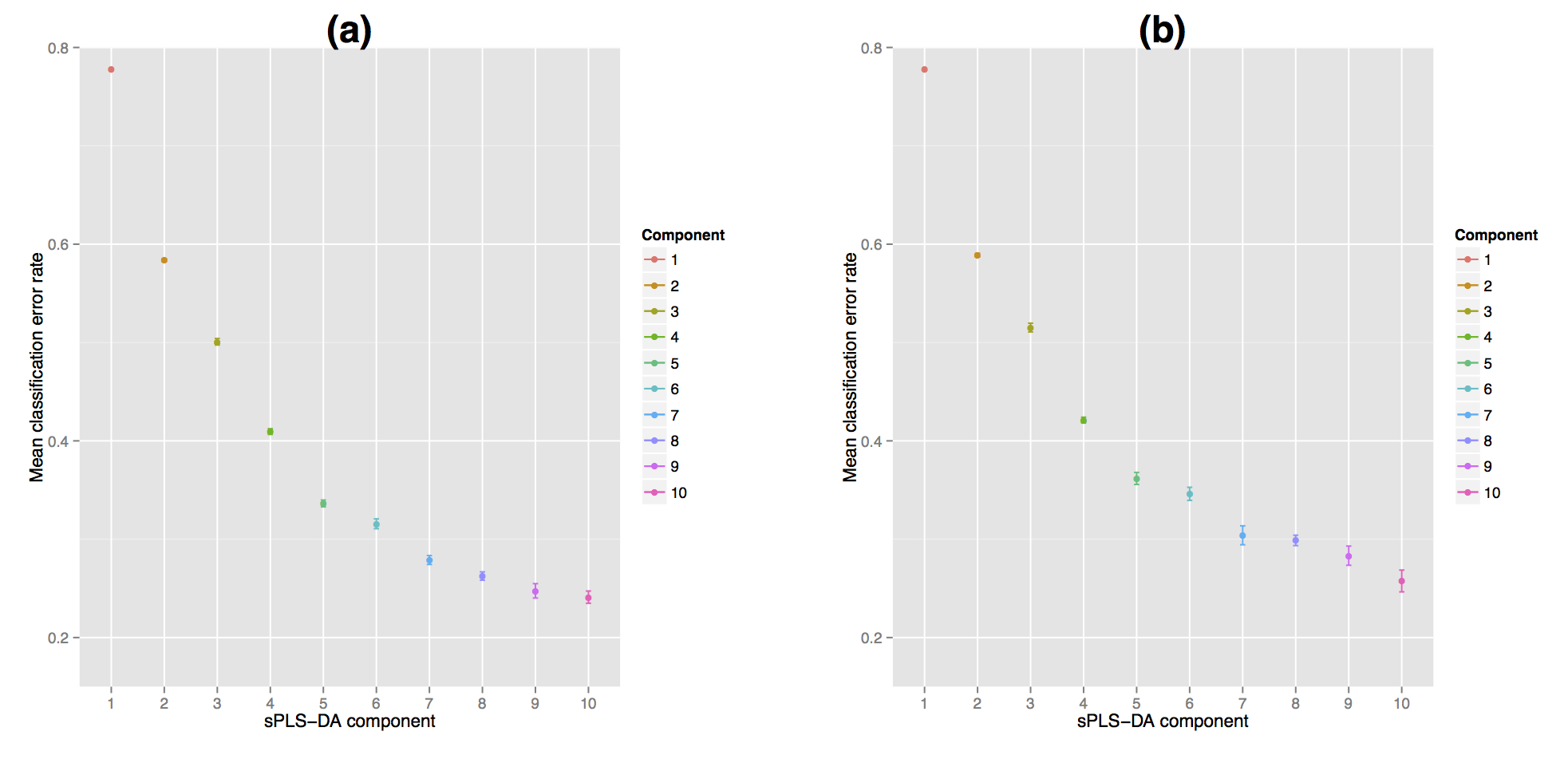

Supplement: S5 Fig — Mean classification performance using 100 * 10-fold cross-validation. Each component is based on an optimal selection of OTU features that leads to the best classification performance. The sPLS-DA classifier was applied on (a) TSS+CLR or (b) CSS normalised data. (TIF) [file pone.0160169.s011.tif]

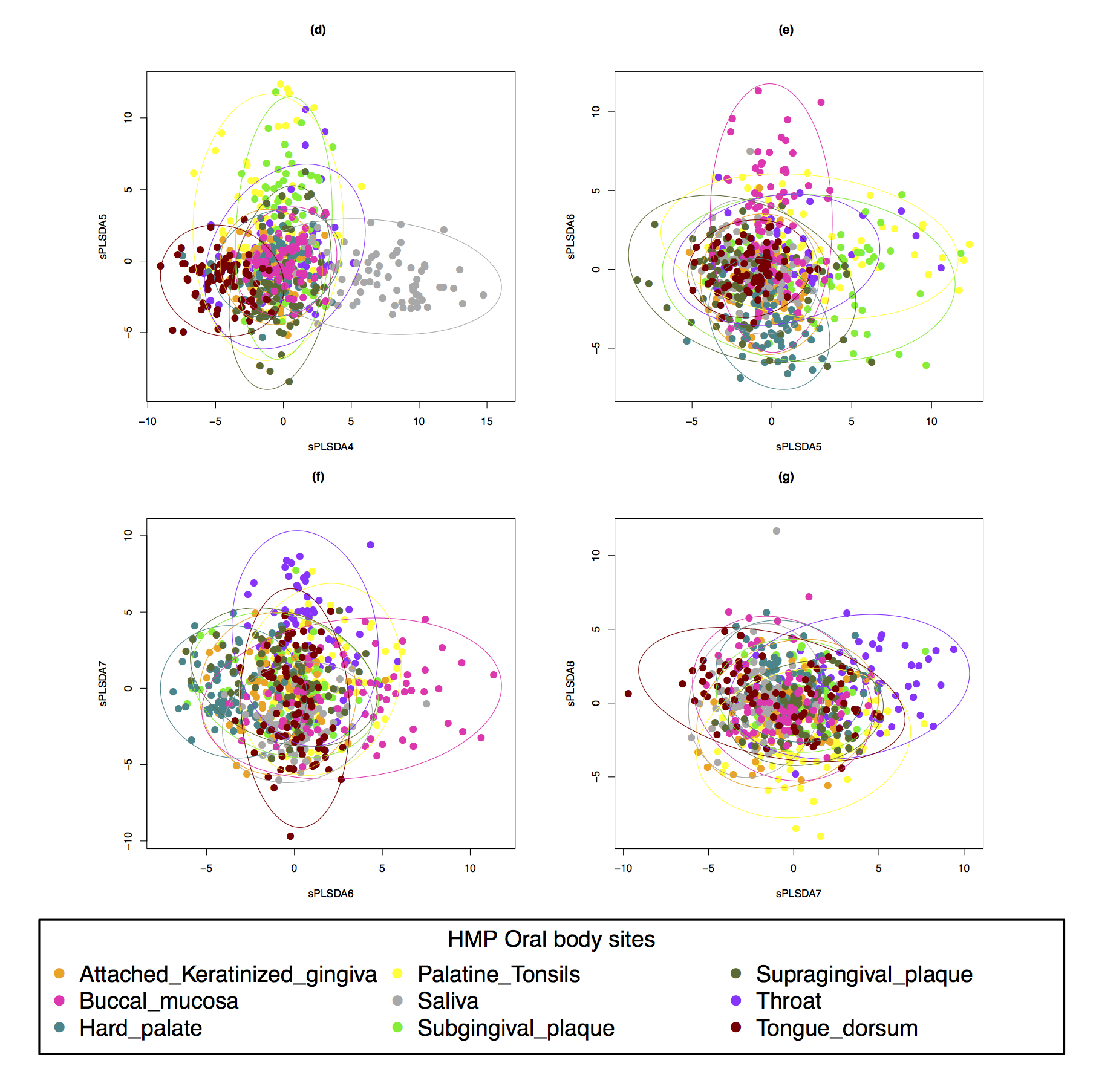

Supplement: S6 Fig — (d) Component 4 vs Component 5, (e) Component 5 vs Component 6, (f) Component 6 vs Component 7, (g) Component 7 vs Component 8. (TIF) [file pone.0160169.s012.tif]

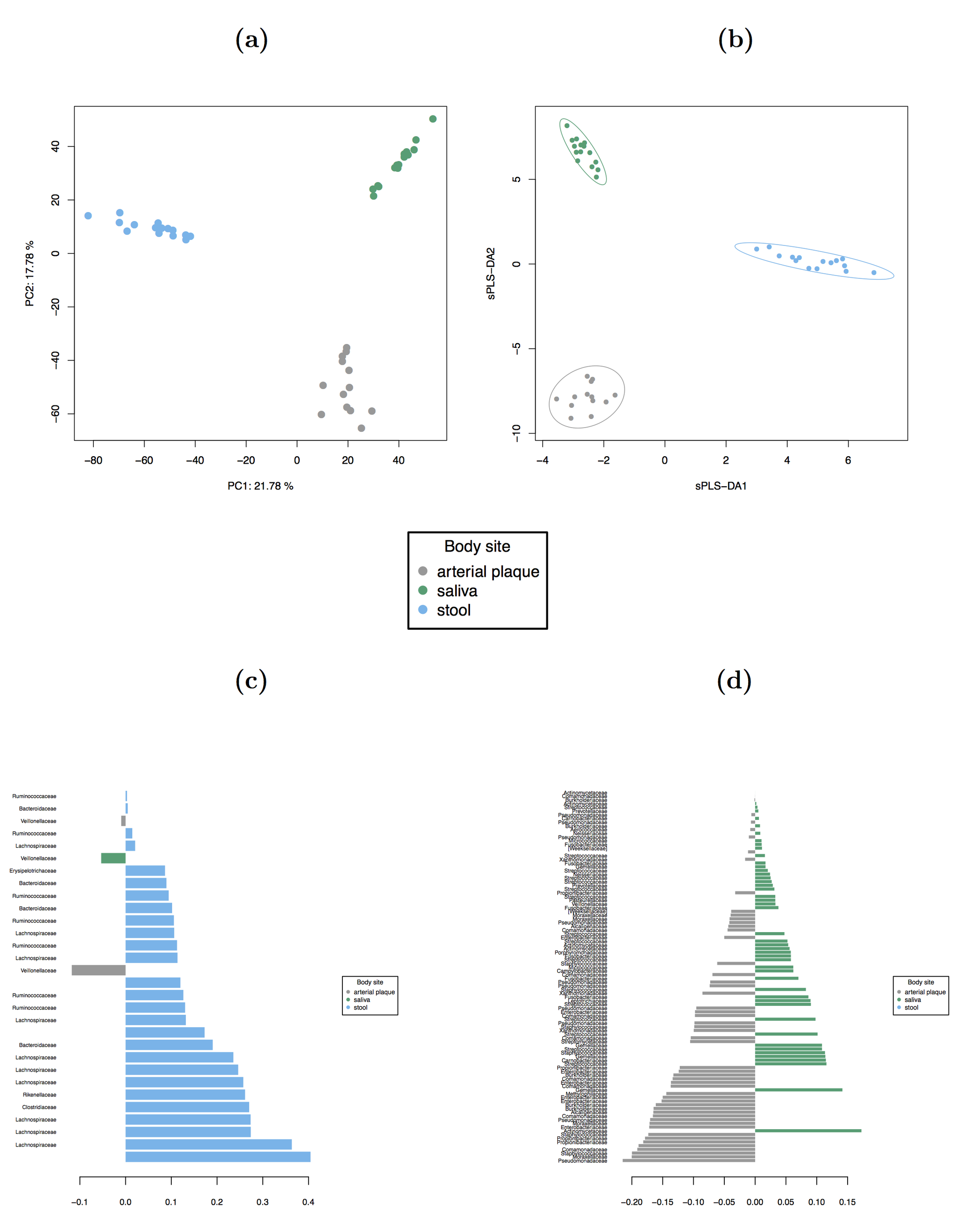

Supplement: S7 Fig — Sample plot on the first two components with (a) PCA (b) sPLS-DA on selected OTU. Contribution plots on the (c) first component (30 OTU selected) and (d) on the second component (100 OTU selected). (TIF) [file pone.0160169.s013.tif]
